# Supplementary material for: The phased pan-genome of tetraploid European potato
Source: Nature. 2025 Apr 16;642(8067):389–97. doi: 10.1038/s41586-025-08843-0 (PMC12158759; doi:10.1038/s41586-025-08843-0)
Supplement: Supplementary file 5 — Supplementary Figs. 3–14, 20, 21, 23–27 and 34–44. [file 41586_2025_8843_MOESM5_ESM.zip › suppl_figure_21/suppl_figure_21i_chr09.pdf]

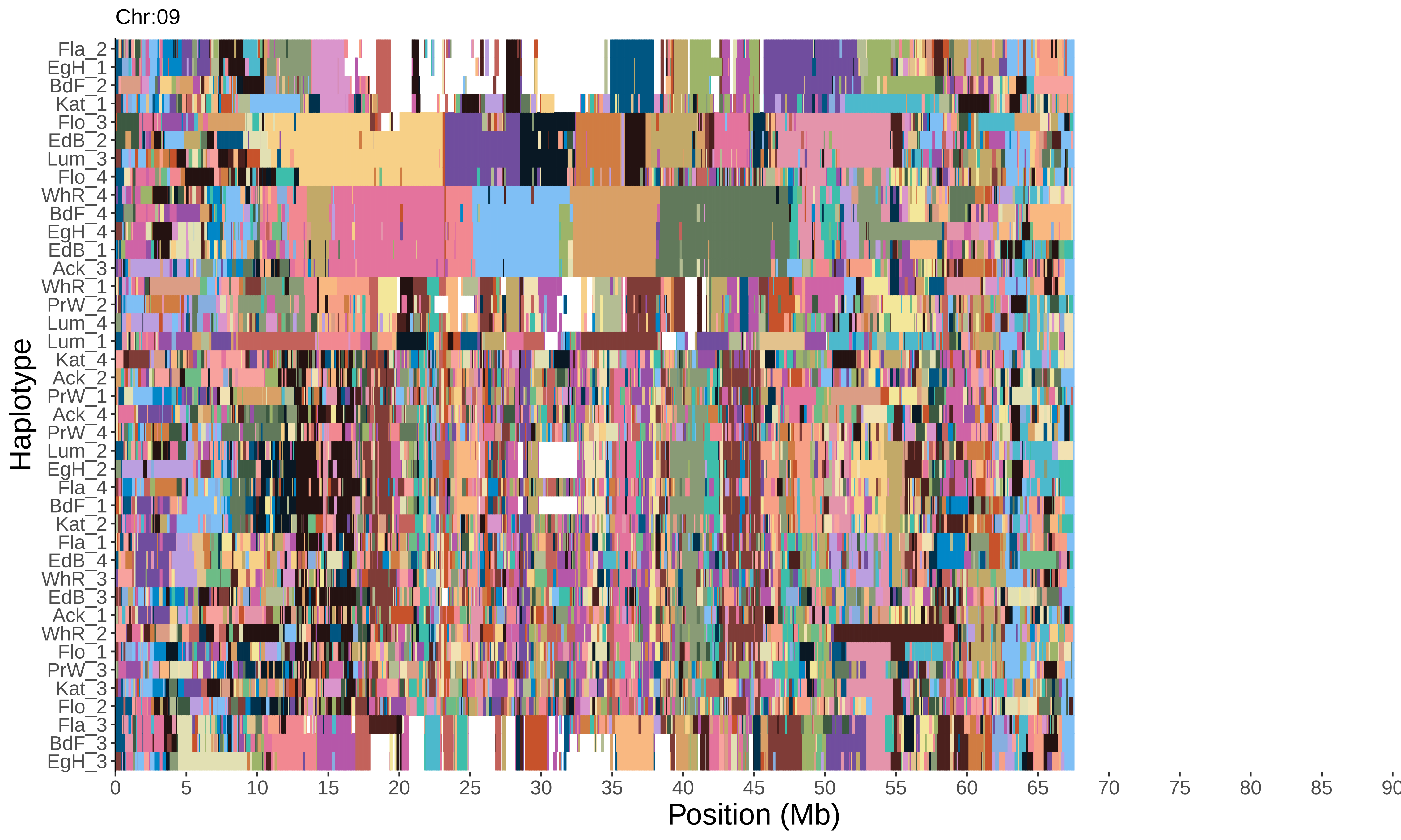
Chr:09
Haplotype
Fla_2
EgH_1
BdF_2
Kat_1
Flo_3
EdB_2
Lum_3
Flo_4
WhR_4
BdF_4
EgH_4
EdB_1
Ack_3
WhR_1
PrW_2
Lum_4
Lum_1
Kat_4
Ack_2
PrW_1
Ack_4
PrW_4
Lum_2
EgH_2
Fla_4
BdF_1
Kat_2
Fla_1
EdB_4
WhR_3
EdB_3
Ack_1
WhR_2
Flo_1
PrW_3
Kat_3
Flo_2
Fla_3
BdF_3
EgH_3
0 5 10 15 20 25 30 35 40 45 50 55 60 65 70 75 80 85 90
Position (Mb)
